# Meta‐analysis of chest CT features of patients with COVID‐19 pneumonia

**DOI:** 10.1002/jmv.26218

**Published:** 2020-07-11

**Authors:** Ying Zheng, Ling Wang, Suqin Ben

**Affiliations:** ^1^ Department of Respiratory and Critical Care Medicine, Shanghai General Hospital Shanghai Jiao Tong University School of Medicine Shanghai China

**Keywords:** chest CT manifestation, COVID‐19 pneumonia, meta‐analysis

## Abstract

The objective of this paper is to perform a meta‐analysis regarding the chest computed tomography (CT) manifestations of coronavirus disease‐2019 (COVID‐19) pneumonia patients. PubMed, Embase, and Cochrane Library databases were searched from 1 December 2019 to 1 May 2020 using the keywords of “COVID‐19 virus,” “the 2019 novel coronavirus,” “novel coronavirus,” and “COVID‐19.” Studies that evaluated the CT manifestations of common and severe COVID‐19 pneumonia were included. Among the 9736 searched results, 15 articles describing 1453 common patients and 697 severe patients met the inclusion criteria. Based on the CT images, the common patients were less frequent to exhibit consolidation (odds ratio [OR] = 0.31), pleural effusion (OR = 0.19), lymphadenopathy (OR = 0.17), crazy‐paving pattern (OR = 0.22), interlobular septal thickening (OR = 0.27), reticulation (OR = 0.20), traction bronchiectasis (OR = 0.40) with over two lobes involved (OR = 0.07) and central distribution (OR = 0.18) while more frequent to bear unilateral pneumonia (OR = 4.65) involving one lobe (OR = 13.84) or two lobes (OR = 6.95) when compared with severe patients. Other CT features including ground‐glass opacities (*P* = .404), air bronchogram (*P* = .070), nodule (*P* = .093), bronchial wall thickening (*P* = .15), subpleural band (*P* = .983), vascular enlargement (*P* = .207), and peripheral distribution (*P* = .668) did not have a significant association with the severity of the disease. No publication bias among the selected studies was suggested (Harbord's tests, *P* > .05 for all.) We obtained reliable estimates of the chest CT manifestations of COVID‐19 pneumonia patients, which might provide an important clue for the diagnosis and classification of COVID‐19 pneumonia.

## Highlights

- We systematically assessed the chest CT manifestations in different severity of COVID‐19 pneumonia.
- We analyzed the CT manifestations comprehensively and systematically from three aspects, including CT features, the number of lobes involved, as well as location and distribution of lesions.
- The sample is large and the conclusions are convincing.

## 1 INTRODUCTION

Since 1st December 2019, a cluster of pneumonia of unknown etiology, now known as coronavirus disease‐2019 (COVID‐19), has been reported in Wuhan, Hubei province, China.^1^ The disease has developed a severe pandemic affecting over 200 countries, areas or territories. According to the data from the World Health Organization (WHO), as of 4 May 2020, more than 3 million cases worldwide have been confirmed with over 20 thousand deaths. Here, we work to synthesize the associated literature by meta‐analysis to describe the chest computed tomography (CT) characteristics of common patients and severe patients with COVID‐19 pneumonia.

## 2 MATERIALS AND METHODS

This meta‐analysis was carried out in accordance with the Preferred Reporting Items for Systematic Reviews and Meta‐Analyses guidelines.^2^ The primary procedures were as follows.

### 2.1 Selection strategy

We conducted a search on PubMed, Embase, and Cochrane Library databases for articles published between 1st December 2019 and 1 May 2020, using the following keywords: “COVID‐19 virus,” “the 2019 novel coronavirus,” “novel coronavirus,” and “COVID‐19.”

### 2.2 Selection criteria

The inclusion criteria for the meta‐analysis were as follows: (a) studies on adult patients with laboratory‐confirmed COVID‐19 pneumonia; (b) studies reported CT feature of patients with various disease severity; and (c) the classification of COVID‐19 was based on the National Guidelines of China (trial version 5).^3^ Patients were divided into four types based on Chinese guideline, including mild, common, severe, and critical severe types. Mild type is defined as clinical symptoms without imaging manifestations of pneumonia. The common type is defined as fever, respiratory symptoms, and imaging manifestations of pneumonia. Severe type is defined as one of the following: (a) respiratory distress with respiratory frequency ≥ 30/min; (b) transcutaneous oxygen saturation ≤ 93% in the rest state; (c) oxygenation index (PaO_2_/FiO_2_) ≤ 300 mm Hg. Critical severe type is defined as one of the following: respiratory failure needing mechanical ventilation, shock, or combination with other organ failure needing ICU intensive care. In our research, the common group included common type patients. The severe group included severe and critical type patients. (a) No limits of language and region; and (b) randomized controlled trials, nonrandomized controlled trials, cohort studies, and cross‐sectional studies on the chest CT manifestation of patients with COVID‐19 pneumonia.

The exclusion criteria were as follows: (a) letters, comments, and reviews; and (b) articles that described fewer than 10 patients.

### 2.3 Data extraction

We reviewed the titles, abstracts, and full texts of manuscripts by duplicate removal based on the above‐mentioned selection criteria. Abstracts of identified articles were separately reviewed by two readers. After we confirmed the inclusion of associated documents, we independently extracted following variables, including the name of the first author, publication year, age of patients, number of patients, and study area. All included literatures were evaluated using the Newcastle‐Ottawa Scale.^4^ The highest quality of the literature is 10 scores and the lowest is 0 score. Data extraction and quality assessment were carried out independently by two reviewers. In case of disagreement, consensus was reached by discussing with a third reviewer.

### 2.4 Statistical analysis

All the statistical analyses were carried out using Stata statistical software version 12.0. The proportions of various CT features in each group were analyzed as follows: original data were transformed by double arcsine method in Stata at first and the final conclusions were drawn using restoring formula (*P* = (sin(tp/2))^2^). The association between the CT features and the severity of COVID‐19 pneumonia was assessed in the form of odds ratio (OR) at a 95% confidence interval (95% CI). Heterogeneity among each study was evaluated using Cochran's *Q* test and Inconsistency index (*I* ^2^) test.^5^ *I* ^2^ > 50% indicates the apparent heterogeneity between the studies and the random‐effects model (Der Simonian and Laird method) would be adopted. Otherwise, the fixed‐effect model (Mantel‐Haenszel model) would be used. Publication bias was assessed for CT characteristics that included more than 10 studies using funnel plots and Harbord's tests. Deviation from the funnel‐shaped distribution of eligible research works suggested the presence of publication bias.

## 3 RESULTS

### 3.1 Inclusion of studies

**Figure 1:**
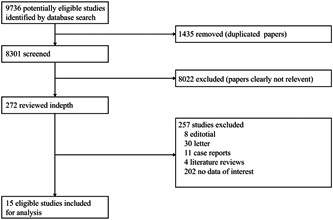
Summary of article selection process

From the databases mentioned above, we retrieved 9736 articles. After removing 1435 duplicated articles, 8301 articles remained. After reading the titles and abstracts, 8022 papers were excluded. After the reading the full text, we kept 15 descriptive studies including 2451 COVID‐19 pneumonia patients in this meta‐analysis.^6^, ^7^, ^8^, ^9^, ^10^, ^11^, ^12^, ^13^, ^14^, ^15^, ^16^, ^17^, ^18^, ^19^, ^20^ The entire process is shown in Figure 1. All the included studies were retrospective studies. The primary characteristics of the literature are exhibited in Table 1. Generally speaking, these articles were considered to be of good quality. All the 15 articles were over 5 scores.

**Table 1:** The characteristics of the literatures

First author, year	Common patients	Severe patients	Research type	Country	Literature quality
K.C. Liu, 2020	46	24	Retrospective study	China	6
Y.H. Xu, 2020	28	13	Retrospective study	China	6
W. Zhao, 2020	79	14	Retrospective study	China	7
Q. Zhong, 2020	35	29	Retrospective study	China	6
K. H. Li, 2020	58	25	Retrospective study	China	7
J. J. Zhang, 2020	77	57	Retrospective study	China	6
P. J. Lyu, 2020	12	39	Retrospective study	China	7
R. Zhang, 2020	84	30	Retrospective study	China	6
W. Zhao, 2020	73	45	Retrospective study	China	6
F. Zheng, 2020	131	30	Retrospective study	China	6
Q. Q. Chen, 2020	100	42	Retrospective study	China	6
J. Wang, 2020	73	20	Retrospective study	China	6
K. B. Cheng, 2020	272	169	Retrospective study	China	7
L. Huang, 2020	58	45	Retrospective study	China	6
Y. Feng, 2020	327	115	Retrospective study	China	7

### 3.2 Meta‐analysis

#### 3.2.1 CT features

The findings of this meta‐analysis indicated that the predominant CT features of common groups were vascular enlargement 0.79 (95% CI, 0.74, 0.84), ground‐glass opacities (GGOs) 0.78 (95% CI, 0.64, 0.89), subpleural bands 0.58 (95% CI, 0.12, 0.97), and interlobular septal thickening 0.51 (95% CI, 0.26, 0.76). Consolidation 0.34 (95% CI, 0.21, 0.48), traction bronchiectasis 0.31 (95% CI, 0.12, 0.55), nodule 0.27 (95% CI, 0.02, 0.65), air bronchogram 0.25 (95% CI, 0.01, 0.64), crazy‐paving pattern 0.24 (95% CI, 0.14, 0.34), reticulation 0.19 (95% CI, 0.02, 0.46), bronchial wall thickening 0.13 (95% CI, 0.04, 0.26), pleural effusion 0.03 (95% CI, 0.00, 0.07), and lymphadenopathy 0.01 (95% CI, 0.00, 0.03) were relatively rare in the common group. All the data above are shown in Table 2.

**Table 2:** The incidences of various CT features in common patients

	95% CI	*P* (Cochran's *Q* test)	*I* ^2^	Number of studies
GGOs	0.78 (0.64, 0.89)	.00	96.35	13
Consolidation	0.34 (0.21, 0.48)	.00	94.98	11
Pleural effusion	0.03 (0.00, 0.07)	.00	77.75	9
Air bronchogram	0.25 (0.01, 0.64)	.00	92.55	3
Lymphadenopathy	0.01 (0.00, 0.03)	.38	5.52	6
Nodule	0.27 (0.02, 0.65)	.00	97.00	3
Crazy‐paving pattern	0.24 (0.15, 0.34)	.00	72.17	6
Interlobular septal thickening	0.51 (0.26, 0.76)	.00	89.78	4
Bronchial wall thickening	0.13 (0.04, 0.26)	.00	84.93	4
Reticulation	0.19 (0.02, 0.46)	.00	96.04	4
Subpleural bands	0.58 (0.12, 0.97)	.00	98.36	3
Traction bronchiectasis	0.31 (0.12, 0.55)	.00	94.41	4
Vascular enlargement	0.79 (0.74, 0.84)	.16	44.84	3
Right upper lobe	0.49 (0.16, 0.83)	.00	95.18	3
Right middle lobe	0.47 (0.23, 0.72)	.00	90.59	3
Right lower lobe	0.80 (0.74, 0.86)	.00	88.92	3
Left upper lobe	0.61 (0.22, 0.93)	.00	96.15	3
Left lower lobe	0.81 (0.53, 0.98)	.00	92.79	3
Unilateral pneumonia	0.22 (0.12, 0.33)	.00	80.83	6
Peripheral	0.91 (0.87, 0.94)	.02	70.47	4
Central	0.05 (0.00, 0.24)	.00	94.79	3
1 lobe involved	0.26 (0.07, 0.52)	.00	90.67	3
2 lobes involved	0.21 (0.01, 0.54)	.00	94.51	3
Over 2 lobes involved	0.57 (0.23, 0.87)	.00	94.88	3

Abbreviations: CI, confidence interval; CT, computed tomography; GGO, ground‐glass opacity.

As Table 3 shows, among severe patients, the predominant CT features included vascular enlargement 0.93 (95% CI, 0.75, 1.00), GGOs 0.82 (95% CI, 0.68, 0.92), interlobular septal thickening 0.80 (95% CI, 0.64, 0.93), air bronchogram 0.67 (95% CI, 0.57, 0.78), consolidation 0.61 (95% CI, 0.42, 0.78), subpleural bands 0.61 (95% CI, 0.10, 1.00), crazy‐paving pattern 0.59 (95% CI, 0.42, 0.79), and traction bronchiectasis 0.52 (95% CI, 0.30, 0.73). Severe patients were less likely to have bronchial wall thickening 0.47 (95% CI, 0.19, 0.77), reticulation 0.46 (95% CI, 0.23, 0.71), pleural effusion 0.19 (95% CI, 0.13, 0.26), nodule 0.18 (95% CI, 0.02, 0.41), and lymphadenopathy 0.07 (95% CI, 0.01, 0.18).

**Table 3:** The incidences of various CT features in severe patients

	95% CI	*P* (Cochran's *Q* test)	*I* ^2^	Number of studies
GGOs	0.82 (0.68, 0.92)	.00	91.91	13
Consolidation	0.61 (0.42, 0.78)	.00	93.86	11
Pleural effusion	0.19 (0.13, 0.26)	.04	50.48	9
Air bronchogram	0.67 (0.57, 0.78)	.35	5.33	3
Lymphadenopathy	0.07 (0.01, 0.18)	.00	75.86	6
Nodule	0.18 (0.02, 0.41)	.01	80.20	3
Crazy‐paving pattern	0.59 (0.42, 0.76)	.00	79.74	6
Interlobular septal thickening	0.80 (0.64, 0.93)	.03	66.96	4
Bronchial wall thickening	0.47 (0.19, 0.77)	.00	89.32	4
Reticulation	0.46 (0.23, 0.71)	.00	84.85	4
Subpleural bands	0.61 (0.10, 1.00)	.00	95.31	3
Traction bronchiectasis	0.52 (0.30, 0.73)	.00	79.77	4
Vascular enlargement	0.93 (0.75, 1.00)	.02	73.25	3
Right upper lobe	0.89 (0.79, 0.96)	.63	0.00	3
Right middle lobe	0.86 (0.76, 0.94)	.23	32.76	3
Right lower lobe	0.98 (0.93, 1.00)	.34	6.93	3
Left upper lobe	0.92 (0.83, 0.98)	.53	0.00	3
Left lower lobe	0.99 (0.95, 1.00)	.66	0.00	3
Unilateral pneumonia	0.05 (0.02, 0.10)	.16	37.45	6
Peripheral	0.88 (0.62, 1.00)	.00	91.56	4
Central	0.17 (0.00, 0.63)	.00	94.68	3
1 lobe involved	0.01 (0.00, 0.05)	.19	39.89	3
2 lobes involved	0.04 (0.00, 0.10)	.50	0.00	3
Over 2 lobes involved	0.94 (0.88, 0.99)	.18	42.15	3

Abbreviations: CI, confidence interval; CT, computed tomography; GGO, ground‐glass opacity.

**Figure 2:**
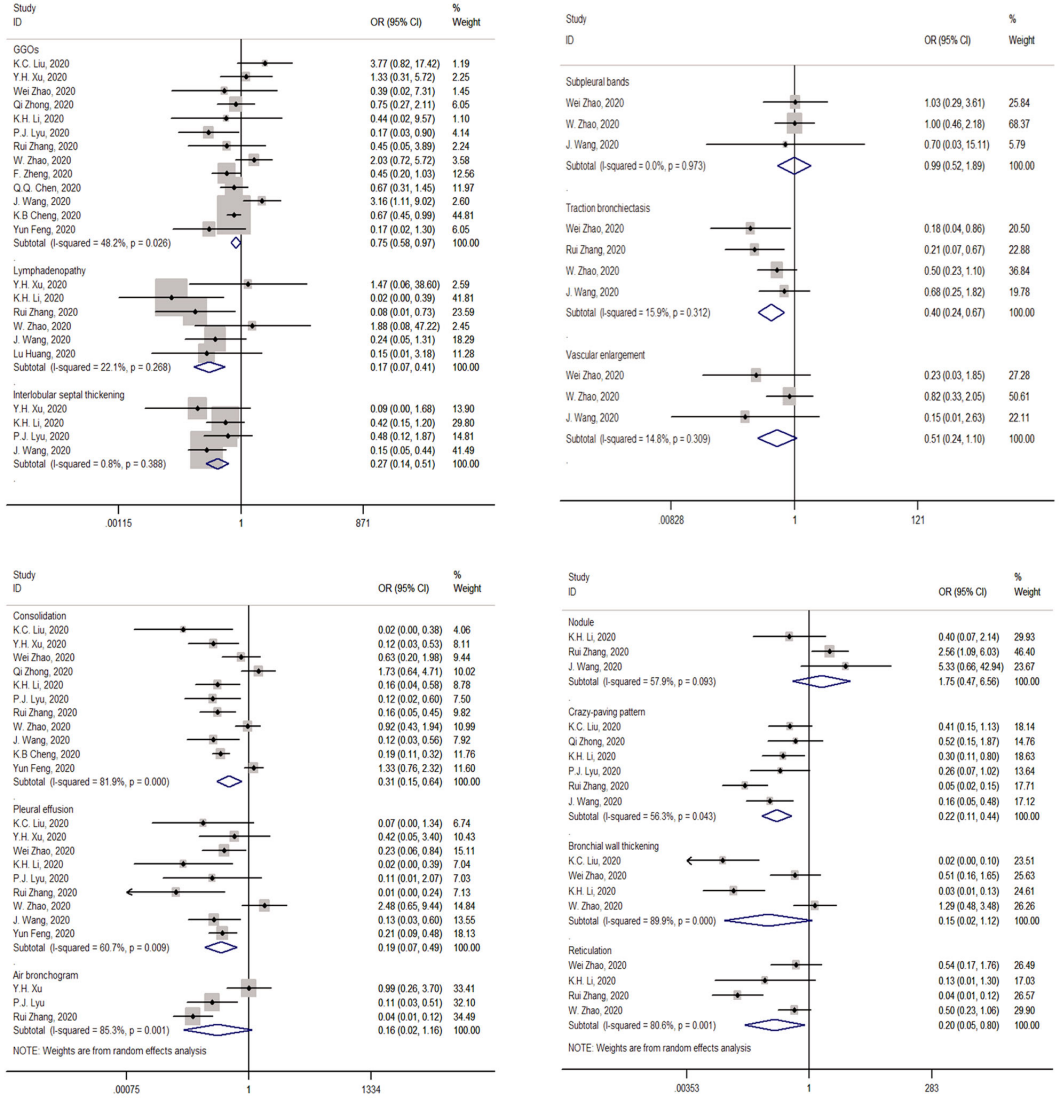
Forest plots of studies on association between computed tomography patterns of common and severe patients

Seven CT features showed significant links with the severity of the disease. Common patients were less frequent to show the following features: traction bronchiectasis (OR = 0.40; 95% CI = 0.24‐0.67; *P* = .002), consolidation (OR = 0.31; 95% CI = 0.15‐0.64; *P* = .001), interlobular septal thickening (OR = 0.27; 95% CI = 0.14‐0.51; *P* = .000), crazy‐paving pattern (OR = 0.22; 95% CI = 0.11‐0.44; *P* = .000), reticulation (OR = 0.20; 95% CI = 0.05‐0.80; *P* = .023), pleural effusion (OR = 0.19; 95% CI = 0.07‐0.49; *P* = .001), and lymphadenopathy (OR = 0.17; 95% CI = 0.07‐0.41; *P* = .008). The remaining six features did not exhibit an apparent association with the severity of disease: nodule (OR = 1.75; 95% CI = 0.47‐6.56; *P* = .093), subpleural bands (OR = 0.99; 95% CI = 0.52‐1.89; *P* = .983), GGOs (OR = 0.75; 95% CI = 0.58‐0.97; *P* = .404), vascular enlargement (OR = 0.51; 95% CI = 0.24‐1.10; *P* = .207), air bronchogram (OR = 0.16; 95% CI = 0.02‐1.16; *P* = .070), and bronchial wall thickening (OR = 0.15; 95% CI = 0.02‐1.12; *P* = .064). All these data are illustrated in Figure 2.

#### 3.2.2 The number of lobes involved

**Figure 3:**
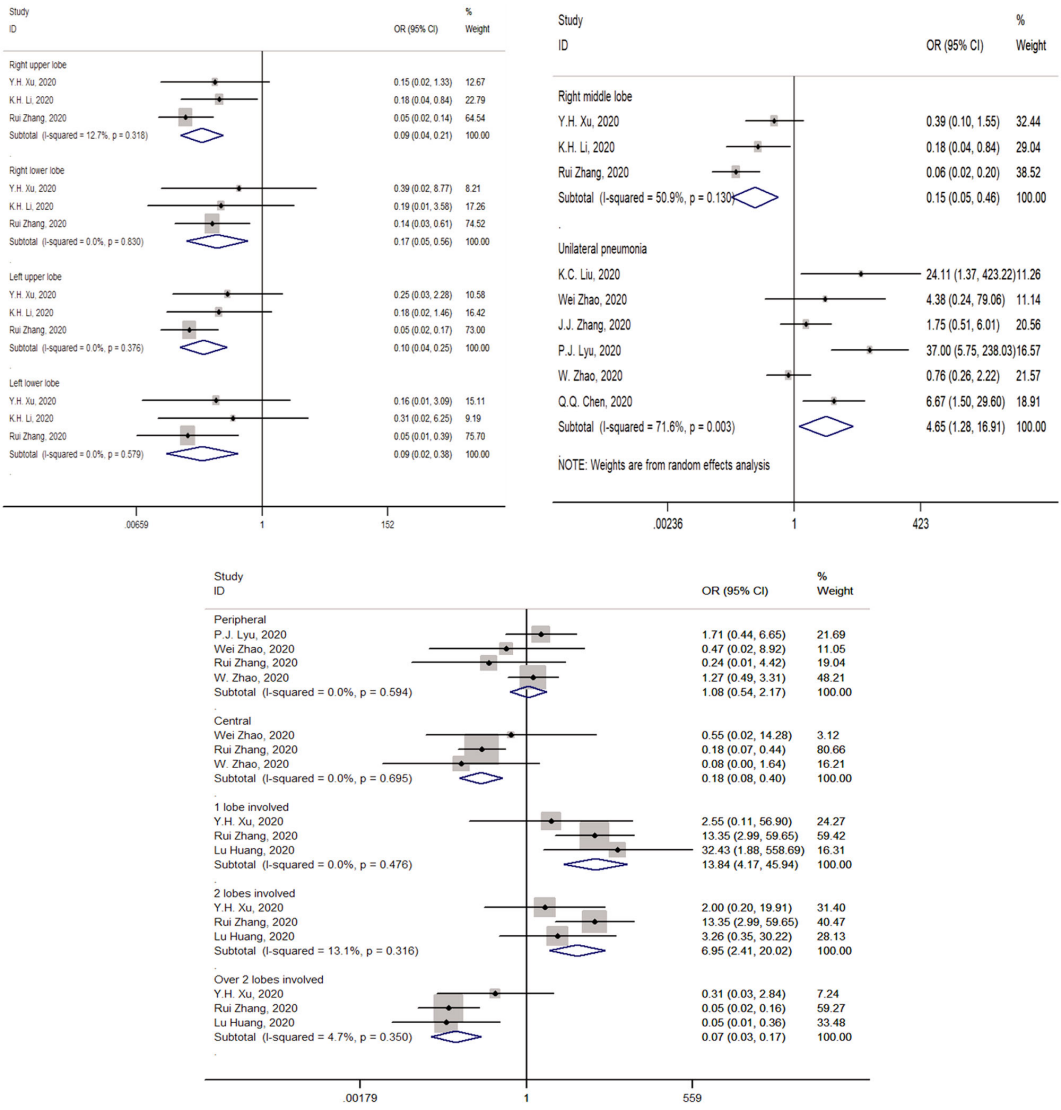
Forest plots of studies on association between lesion locations of common and severe patients

The pooled incidences of one lobe affected, two lobes affected, and over two lobes affected in common patients were, respectively, 0.26 (95% CI, 0.07‐0.52), 0.21 (95% CI, 0.01‐0.54), and 0.57 (95% CI, 0.23‐0.87) (Table 2). The pooled incidences of one lobe affected, two lobes affected, and over two lobes affected in severe group were, respectively, 0.01 (95% CI, 0.00‐0.05), 0.04 (95% CI, 0.00‐0.10), and 0.94 (95% CI, 0.88‐0.99) (Table 3). Compared with severe patients, common patients were more likely to have radiographic abnormalities with one lobe involved (OR = 13.84; 95% CI = 4.17‐45.94; *P* = .000) and two lobes involved (OR = 6.95; 95% CI = 2.41‐20.02; *P* = .004). They were less likely to have abnormalities with over two lobes involved (OR = 0.07; 95% CI = 0.03‐0.17; *P* = .000) (Figure 3).

#### 3.2.3 Location and distribution of lesions

In common patients, the pooled incidences of unilateral pneumonia, right upper lobe involved, right middle lobe involved, right lower lobe involved, left upper lobe, left lower lobe, peripheral distribution, and central distribution were, respectively, 0.22 (95% CI, 0.12, 0.33), 0.49 (95% CI, 0.16, 0.83), 0.47 (95% CI, 0.23, 0.72), 0.80 (95% CI, 0.74, 0.86), 0.61 (95% CI, 0.22, 0.93), 0.81 (95% CI, 0.53, 0.98), 0.91 (95% CI, 0.87, 0.94), 0.05 (95% CI, 0.00, 0.24) (Table 2). In severe patients, the pooled incidences of unilateral pneumonia, right upper lobe involved, right middle lobe involved, right lower lobe involved, left upper lobe, left lower lobe, peripheral distribution and central distribution were, respectively, 0.05 (95% CI, 0.02, 0.10), 0.89 (95% CI, 0.79, 0.96), 0.86 (95% CI, 0.76, 0.94), 0.98 (95% CI, 0.93, 1.00), 0.92 (95% CI, 0.83, 0.98), 0.99 (95% CI, 0.95, 1.00), 0.88 (95% CI, 0.62, 1.00), 0.17 (95% CI, 0.00, 0.63) (Table 3).

Compared with severe patients, common patients were less frequent to show abnormalities at the following locations: right upper lobe (OR = 0.09; 95% CI = 0.04‐0.21; *P* = .000), right middle lobe (OR = 0.14; 95% CI = 0.06‐0.29; *P* = .001), right lower lobe (OR = 0.17; 95% CI = 0.05‐0.56; *P* = .005), left upper lobe (OR = 0.10; 95% CI = 0.04‐0.25; *P* = .000), left lower lobe (OR = 0.09; 95% CI = 0.02‐0.38; *P* = .002), and central distribution (OR = 0.18; 95% CI = 0.08‐0.40; *P* = .000). Peripheral distribution did not show a significant association with the severity of disease: (OR = 1.17; 95% CI = 0.56‐2.44; *P* = .668). Common patients were more frequent to have unilateral pneumonia: (OR = 4.65; 95% CI = 1.28‐16.91; *P* = .020) (Figure 3).

### 3.3 Publication bias

**Figure 4:**
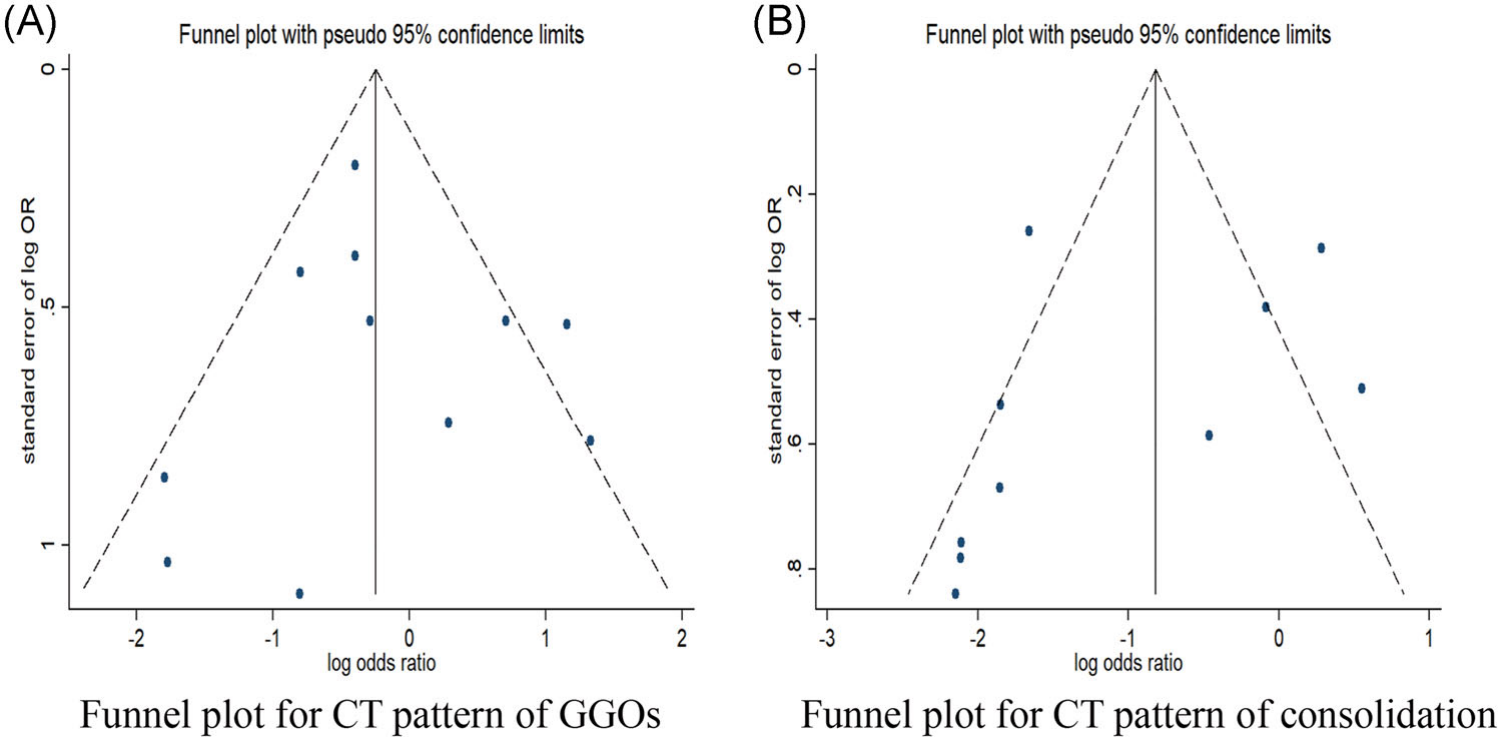
Funnel plot for publication bias

Publication bias was tested for the CT features of GGOs (n = 13) and consolidation (n = 11). No significant publication bias was suggested in GGOs with either Harbord's test (*P* = .885) or funnel plot. Similarly, no significant publication bias was suggested in consolidation with either Harbord's test (*P* = .348) or funnel plot (Figure 4).

## 4 DISCUSSION

Coronaviridae (CoVs) are well‐known single‐stranded RNA viruses that are ubiquitous in many mammals including humans.^21^ According to the antigenic criteria and phylogenetic analyses, they are categorized into three groups: alpha‐CoVs, beta‐CoVs, and gamma‐CoVs.^22^ Even though most human CoVs usually result in mild infections, two beta‐CoVs of them, SARS‐CoV and MERS‐CoV, have been known to lead to serious outbreaks and caused a large amount of cumulative cases in the past.^23^, ^24^ The already identified CoVs might be a small part of the family Coronaviridae and there are many other severe and novel viruses to be revealed. Very recently, “pneumonia of unknown etiology” emerged in Wuhan, Hubei, China. Deep sequencing analysis of samples in respiratory tract confirmed that the cause of the pneumonia was a novel beta‐CoVs coronavirus (COVID‐19 virus). Previous studies have suggested that COVID‐19 virus possibly originated in bats as the virus is 96% identical with a bat coronavirus at the whole‐genome level.^25^ Researchers have reported that the virus can transmit from human to human as well as spread from patients to medical workers,^26^, ^27^ but the specific transmission routine of COVID‐19 virus among hosts is still unclear.

COVID‐19 virus can be transmitted mainly through droplets and contact. The rapid infection and high incidence contribute to far‐reaching public health ramifications.^26^ Chest CT is an important screening tool of the diagnostic workup for COVID‐19 pneumonia because of its high sensitivity and convenience.

In the present meta‐analysis, we found that GGOs, vascular enlargement, interlobular septal thickening, and subpleural bands were the most common findings in either common or severe patients. Compared to those of common patients, some CT manifestations were more frequent in severe patients, such as traction bronchiectasis, interlobular septal thickening, consolidation, crazy‐paving pattern, reticulation, pleural effusion, and lymphadenopathy. These differences were firmly related to development at different stages. At the early stage of COVID‐19, the virus invades and replicates in the alveolar epithelium, resulting in the alveolar cavity to leak with the distributions mainly under the pleural or around the peribronchovascular regions, the involved lesions usually manifest as localized GGOs, subpleural band, vascular enlargement, and peripheral distribution on CT.^28^, ^29^ With the disease progresses, the range of involved alveoli and mucosa increases, the bronchial wall swells, which contributes to the patterns of air bronchograms with consolidation and bronchial wall thickening.^30^ The patterns of crazy‐paving, interlobular septal thickening, and reticulation basically reflect the involvement of pulmonary interstitium, such as interlobular interstitial edema. Severe patients also showed apparent lymphadenopathy and pleural effusion. These extrapulmonary manifestations may indicate the progression of the disease and the occurrence of deteriorated inflammation. Lesions mainly occurred in peripheral area and most COVID‐19 pneumonia patients exhibited the bilateral abnormalities. In common patients, lower lobes were involved more frequently than the upper and middle lobes. Except for peripheral distribution and multilobar involvement, posterior involvement is another important characteristic of lesions distribution.^31^

COVID‐19 pneumonia should be identified from other viral pneumonia caused by severe acute respiratory syndrome‐related coronavirus (SARS‐CoV), Middle East respiratory syndrome‐related coronavirus (MERS‐CoV), influenza viruses, adenovirus, respiratory syncytial virus, and so forth. The H1N1 pneumonia is typically marked by scattered GGOs or consolidations in peribronchovascular or subpleural distribution.^32^ Compared with the immunocompromised population, small airway abnormalities such as airway thickening and dilatation, centrilobular nodules, and tree‐in‐bud sign are rare in immunocompetent patients.^32^ What is more, some patterns including lymphadenopathy and pleural effusions are usually absent in H1N1 pneumonia.^32^ For H7N9 pneumonia, the most common findings on CT are GGOs.^33^ Diffused consolidations, air bronchograms, and interlobular septal thickening are the second most common imaging abnormalities.^33^ Besides, H7N9 pneumonia usually progresses rapidly and the right lower lobe is easier to be involved.^33^ The most common radiographic abnormalities in adenovirus pneumonia are diffuse bilateral bronchopneumonia and lobar atelectasis.^34^ Thickened interlobular septa and diffuse GGOs are infrequent in adenovirus pneumonia.^34^

Different from COVID‐19 pneumonia, adenovirus is much easier to infect pediatric patients. Right upper lobe atelectasis is common in infants, while in older children, atelectasis usually occurs at left lower lobe.^34^ In the respiratory syncytial virus (RSV) infected patients, CT usually manifests as the pattern of nodules, tree‐in‐bud opacity, and bronchial wall thickening.^35^ Compared with other viral pneumonia, consolidation and GGOs are rarely observed in RSV‐infected pneumonia.^35^ Similar with adenovirus, infants and immunocompromised adults are more susceptible to RSV‐infected pneumonia. SARS‐CoV, MERS‐CoV, and COVID‐19 virus all belong to Coronaviridae and they share a lot of similarities in CT manifestation. GGOs and consolidations that mainly distribute at the peripheral lower lung zone are also the predominant abnormalities on CT scanning of patients with SARS and MERS.^36^, ^37^, ^38^ Interlobular septal thickening and intralobular lines are common as well. What is more, opposite to patients with COVID‐19, patients with SARS and MERS manifest unifocal involvement more often than multifocal involvement on chest CT.^39^ In the early stage, the lesions mainly locate under the pleura, with the progression of illness, lesions become diffuse. After recovery, the fibrotic changes may be irreversible. Some patterns such as mediastinal lymph nodes and substantial effusions are irregular.^37^ Even though there are some traceable differences on chest CT between these viral pneumonias, it is still hard work to distinguish COVID‐19 from other vial pneumonia. Real‐time polymerase chain reaction is needed for a definitive diagnosis.

To the best of our knowledge, this article is the first to systematically assess the chest CT manifestations in different severity of COVID‐19 pneumonia. The analysis is rigorous and the conclusions are convincing. This study also has limitations. First, all the studies are retrospective studies and significant heterogeneity are observed. Second, some studies with small samples were also included in the analysis and the strength of the study may also be limited. Third, all the patients included are Chinese and the conclusions may be less representative.

In conclusion, our results indicate that vascular enlargement and GGOs are common chest CT findings in COVID‐19 pneumonia. Severe patients are more likely to have CT abnormalities with traction bronchiectasis, interlobular septal thickening, consolidation, crazy‐paving pattern, reticulation, pleural effusion, and lymphadenopathy. All five lobes tend to be affected. However, because of the limitations mentioned above, studies with larger sample size and more rigorous design should be carried out.

## CONFLICT OF INTERESTS

The authors declare that there are no conflict of interests.

## AUTHOR CONTRIBUTIONS

SB conceived the study and drafted and revised the manuscript for intellectual content. YZ acquired the data and drafted the manuscript. LW performed the statistical analysis. All the authors approved the final manuscript.
